# Global e-Learning in Early Nutrition and Lifestyle for International Healthcare Professionals: Design and Evaluation of the Early Nutrition Specialist Programme (ENS)

**DOI:** 10.3390/nu13030775

**Published:** 2021-02-27

**Authors:** Brigitte Brands, Ngoc Nhan Tran, Erin Baudendistel-Happ, Marina Sanchez-Garcia, Martin R. Fischer, Berthold Koletzko

**Affiliations:** 1 Department of Paediatrics, Dr. von Hauner Children’s Hospital, University Hospital, LMU—Ludwig-Maximilians-Universität München, 80377 Munich, Germany; Ngoc.Tran@med.uni-muenchen.de (N.N.T.); Erin.Baudendistelhapp@med.uni-muenchen.de (E.B.-H.); Marina.Sanchez@med.uni-muenchen.de (M.S.-G.); Berthold.Koletzko@med.uni-muenchen.de (B.K.); 2 Institute for Medical Education, LMU University Hospitals, LMU—Ludwig-Maximilians-Universität München, 80336 Munich, Germany; Martin.Fischer@med.uni-muenchen.de

**Keywords:** e-learning, healthcare professionals, CME, early nutrition, first 1000 days

## Abstract

Background: Every encounter a healthcare professional has with new or expecting parents offers an opportunity for addressing improved early nutrition and lifestyle. Evidence-based qualification programmes via e-learning offer valuable tools for attenuating the world’s huge double burden of both under- and overnutrition in early childhood. We evaluated use and learner satisfaction of a global e-learning programme on early nutrition and lifestyle addressing international healthcare professionals. Methods: We implemented the Early Nutrition Specialist Programme (ENS) with six interactive e-learning courses on early nutrition building on more than ten years of experience with global e-learning platforms, expert knowledge and an international network in the subject field. We collected descriptive and explorative evaluation data on usage and learner satisfaction with a questionnaire and log data over three years among 4003 learners from 48 countries. Results: Results show high completion of the ENS programme, with 85.5% of learners finalizing the programme after enrollment into the first of six courses. Very good results were provided for learner satisfaction with the courses (96.7% of users), for increasing understanding of the topic (97.4%) and matching the indicated time investment (94.4%). Most predominant themes in the open text fields of user feedback questionnaires were “Increase interactivity or number of audio-visuals”, “Content suggestions or more examples” and “Technical (quality) issues or navigation problems”. Conclusions: The ENS programme evaluation shows high completion rates and level of satisfaction by learners from numerous countries. The different needs for Continuing Medical Education (CME) of healthcare professionals in diverse healthcare system settings can be met by a joint e-learning qualification programme. Further optimizations will be implemented based on user feedback. More research with a learning analytics approach may help to further identify the most effective and efficient didactic and pedagogic elements of e-learning.

## 1. Introduction

For the global population of children born today, the greatest life-long burden for health and quality of life arises from the rapidly increasing prevalence of non-communicable disease (NCD, e.g., obesity, diabetes, cardiovascular diseases, lifestyle-related cancers, and others), and from the increasing sociodemographic disparities of disease risks. A large body of evidence for the impact of Developmental Origins of Adult Health and Disease has been established during the last two decades, demonstrating that events before and during pregnancy, infancy and toddlerhood markedly modify later NCD risk [1,2].

Healthcare Professionals (HCPs), being a key gateway for optimizing nutrition and lifestyle during the critical phase of early development and growth in the first 1000 days of life, are an important target group. HCPs can be reached by effective and efficient lifelong qualification and education programmes. Educational research indicates that implementation of e-learning programmes, also in the field of Continuing Medical Education (CME), offer a variety of benefits such as flexibility with time and space, cost effectiveness, the opportunity to take individual learner differences into account and allow for self-pacing [3,4,5]. However, potential disadvantages for e-learning implementation have also been identified such as lack of learner–teacher/learner–learner interaction, higher risk of plagiarism and high attrition rates [6]. Attrition is influenced by course design. Therefore, emphasis should be placed on using attractive educational designs with highly interactive learning formats when developing e-learning programmes. Capitalizing from nearly ten years of generating and conducting e-learning programmes at a global scale, we developed a highly interactive CME accredited e-learning qualification programme entitled “The Early Nutrition Specialist (ENS)” for international healthcare professionals in three different languages (English, Spanish, French). Here, we report on the evaluation results descriptively and exploratively collected over three global execution cycles from 2018–2020 of the ENS programme in English language in 4003 users from 48 countries.

## 2. Materials and Methods

### 2.1. The Early Nutrition Specialist Programme (ENS): Concept and Programme Execution

The ENS programme, run by the Ludwig-Maximilian-Universität München (LMU Munich, Germany) in collaboration with the LMU University Hospital, Dept. of Paediatrics, Dr. von Hauner Children’s Hospital and the Early Nutrition eAcademy (ENeA), was established and piloted in 2017 with around 600 doctors in practice in three countries of the Middle East (Iran, Egypt, Saudi Arabia). Since then, the programme was expanded and three further global cycles were conducted with more than 5000 healthcare professionals in more than 48 countries worldwide. The ENS programme capitalizes from more than ten years’ experience in conceptualization, implementation and execution of e-learning platforms and courses, including the Early Nutrition eAcademy (ENeA; >12,000 users, 173 countries, 4 languages including English, Mandarin Chinese, Spanish and Turkish), dedicated e-learning programmes addressing Southeast Asia and Southern Africa and others, and an international network of collaborating experts on research and healthcare related to the subject field. The development, implementation and evaluation of the ENeA programmes was supported by several European Union funded grants including the Framework 7 Programme EarlyNutrition.

The ENS programme contains highly interactive online courses using the open-source learning management software MOODLE (Modular Object Oriented Dynamic Learning Environment). Based on extensive expert knowledge in the field of early nutrition and lifestyle, the ENS programme consolidates the current scientific evidence base, international and regional recommendations, together with latest research findings into e-learning courses that are directly relevant for practical application by international healthcare professionals in the field. 

**Figure 1:**
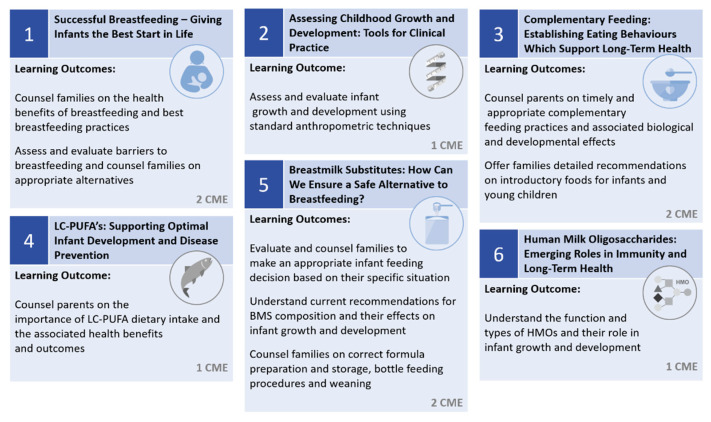
The Early Nutrition Specialist (ENS) Curriculum. Note: LC-PUFA stands for Long Chain Polyunsaturated Fatty Acid.

The learning curriculum contains Core Courses alternating each month with Focus Courses, with a defined start and end date synchronized for all learners and a total duration of six months. While Core Courses provide comprehensive basic knowledge on the specific topic, Focus Courses offer knowledge in specialized areas (Figure 1). The Core Courses consists of three topics each with a learning time of 2 × 45 min plus 15 min per month. The Focus Courses have a learning time of 45 min plus 15 min. Learners can access the ENS programme by self-enrolling with an access code, which consists of a unique 11-digit number located on the personal voucher invitation to the programme. The ENS programme is accredited by the German Medical Association (“Bundesärztekammer”) and the European (UEMS) CME accreditation body, which is also accepted by the American (AMA) CME accreditation authority. At the end of each month, the participant must successfully complete a CME test to collect CME credits. This test consists of ten multiple-choice questions. To pass a CME test and earn credits, learners must achieve a minimum score of 70% correct answers. Every participant has three attempts to pass the test. Regardless of the result of a CME test, the user is still able to continue the ENS programme and collect CME credits in the subsequent courses. At the end of the ENS programme, a final ENS CME Certificate is automatically generated by the platform with the total number of achieved CME credits collected over the whole ENS programme duration. To get the final ENS CME Certificate of Completion, a minimum threshold of five out of nine available CME credits and the completion of all courses is required. The Certificate is issued as an official professional education certificate by LMU Munich and the ENeA.

Learning progress is continuously self-monitorable by the learner and accompanied by an automatic learner management system (automatic and individualized SMS-messages and email reminders and next course announcements are sent to the learner).

### 2.2. Design of the ENS e-Learning Platform and Courses

Didactic and pedagogic approach. Despite a vast amount of experience generated in creating e-learning modules since 2011, one of the key constraints arising from evaluation of the existing ENeA Global platform [7] (https://enea.med.lmu.de/, accessed on 1 December 2020) was the rather low level of interactivity. Effective CME programmes need to disseminate information to practitioners in a way that the user actively assimilates the knowledge. According to constructivist approaches, it is proposed that in the process of knowledge construction, the learner should be an active agent wherever possible [8]. Interactive learning environments may hold the key to not simply transmitting information but enabling learners to actively engage in the process of learning [9]. Thus, the introduction of enhanced interactivity was a key focus during the development of the ENS programme. We selected the open-source HTML5 package (H5P) plugin in MOODLE, which allows mobile-friendly activities such as drag and drop questions, interactive videos and summaries the learner can create themselves and enable the learners to directly employ their gained knowledge [10]. Due to limited personnel capacities for accompanying the ENS programme from the instructor and moderator side throughout its execution phase of six months, we focused on learner–content and learner–interface interactions as modes of interactivity. The e-learning courses were based on the theory of Mayes’ Conceptualisation Cycle, which combines the three major learning theories or perspectives as an iterative cycle of learning [11]. The situational perspective focuses on the motivation of the learner, the associative perspective concentrates on the nature of the accomplishment, and the cognitive perspective examines the function of comprehension on performance. Each of the described perspectives is linked to a specific type of pedagogics. Thus, the following formats were used for generating each e-learning course of the ENS programme to reflect the stages of the learning cycle. In stage one (conceptualisation stage, associative perspective), learners are exposed to novel ideas/concepts. This is achieved through reading lecture notes, watching videos online, etc. During stage two (construction stage, cognitive perspective), new concepts are applied by learners to execute meaningful activities. Tasks involved at this stage are undertaking quizzes, writing a report, etc. In stage three (dialogue stage, situational perspective), the new concepts are usually tested through conversation with peer learners as well as instructors. This feedback facilitates the revision of erroneous concepts and can also be achieved through automatic feedback or feedback on posts in discussion forums. Media types were chosen according to Laurillard’s Conversational Theory of Learning, comprising six different types: narrative, communicative, interactive, productive, adaptive and integrative, which all support the learning process [12]. The entire development process was underpinned by established principles of multimedia e-learning design to encourage the reduction of extraneous cognitive load in the learner, aiming to ensure the most effective learning [13].

Course structure and design elements. Every course starts with a self-assessment test. Completion of this test is a mandatory requirement for accessing the subsequent lessons. The test consists of five questions on the content covered in the respective course. The purpose of this test is to allow the learner to evaluate their pre-existing knowledge on the content, to enhance the motivation for completing the course, and to facilitate monitoring the learning success. A variety of formats were used for the self-assessment questions including drag and drop questions, fill in the blanks, true and false questions and drag the text questions. Interactive videos were incorporated into each of the following up to eight lessons, which have to be followed in the proposed order per course. In-video quizzes are used to test the learners’ recall of the topic directly after exposure to content provided in that lesson [14]. Content recall was tested at the end of each course, where the learner creates their own summary of key statements by selecting options from given statements. Before offering access to the CME multiple choice test at the end of each course, the user is asked to complete an evaluation questionnaire. Throughout the course, each learner can access an interactive forum to exchange knowledge, ask questions or simply network with peer learners. In a dedicated references and download corner of each course, additional information can be accessed. Together, all of these elements were chosen to engage the learner in both behavioural interactivity as well as cognitive interactivity. To facilitate the use for busy clinicians with limited protected time, the platform continuously tracks and saves the progress of the learner, with interruption being possible at any time. 

### 2.3. Descriptive and Explorative Evaluation of the e-Learning Platform and Courses

Descriptive and explorative data were collected from the course participants of each of the three global cycles of the ENS programme in 2018, 2019 and 2020, and data were jointly analysed. No substantial changes in the programme were implemented during this time period except for updating the literature in each course before the 2020 cycle and optimizing technical features of both the platform and the automatic user reminder system. Descriptive user statistics and explorative evaluation questionnaires were analysed in December 2020 using MOODLE inherent statistical and report generation plugins on the ENS e-learning platform. All participants gave active and informed consent to terms and conditions including a detailed description of use of their data when registering on the e-learning platform with opt-in procedures in full compliance with the 2018 European Union General Data Protection Regulation (GDPR) requirements. All processes received clearance by the data protection clearance officer at LMU University Munich.

Explorative analysis. We measured learner satisfaction through the compulsory course evaluation questionnaire that had to be completed at the end of each course before being able to access the CME test and certificate (cf. above). The majority of questions were adapted from Awad et al. (2013). Questions were designed to evaluate learners’ self-perception of learning gains, their satisfaction with the time investment, as well as their affective state after completing the course. A range of question formats and different scaling were utilised to avoid the risk of mechanistic responses [15], including questions with free text answers to gain a further insight into the users’ experiences. The evaluation questions are shared in Appendix A, Table A1. A thematic data analysis technique was used for qualitative analysis of free text responses in the questionnaires of the first three courses. The content of responses was independently categorized into different themes by two coders and then compared amongst each other to reinforce the reliability of the coding. The themes were then qualitatively evaluated to determine the most prominent categories with which the most comments were assigned. 

## 3. Results

### 3.1. Descriptive Analysis: User Statistics

For the three global cycle English version of the ENS programme, a total of 4628 participants registered on the e-learning platform, among which 4003 users have started the first course (initial dropout rate of 13.5%, see Table 1). The majority of participants were paediatricians (83.7%). About 40% of all the participants were between 36 and 45 years of age. Gender was nearly equally represented (female 49.5%, male 49.9%). A large portion of users (39.2%) had more than fifteen years of professional experience. 

In the three global cycles, 4003 users from 48 countries were enrolled in the first course of the English language version of the ENS programme, with the highest number enrolled at the beginning of the programme in the breastfeeding course (*n* = 4003) and a subsequent dropout rate towards the end of the programme of 14.5 %. We obtained information on the number of registered users and country of origin, the number of users enrolled in each course, and the number of CME tests completed per course and their pass-rates (Table 2).

### 3.2. Explorative Evaluation: Learner Satisfaction

The evaluation questionnaires of the English language courses were completed by 3915 users in the first course and 3470 users in the last course, reflecting an overall dropout rate in the English programme version of 11.4%. Since participation in the evaluation questionnaire was fully anonymous, associations with sociodemographic characteristics could not be analyzed. Here, we report on three selected items used in all six courses’ evaluation questionnaires to provide a comparative analysis amongst courses.

English language programme version: The highest number of users agreeing or fully agreeing that the course has increased their understanding of the topic was seen for the “Successful Breastfeeding” course (99.1%), with the lowest percentage giving this rating for the course “Assessing Childhood Growth and Development” (94.6%) (Table 3). The course “Human Milk Oligosaccharides” received the best scoring among all courses for the criterion “The course matched the prescribed time investment” with 96.4% of users agreeing or fully agreeing while this percentage was lowest, although still higher than 90%, for the course “Complementary Feeding” (91.1%). Some 95.5% to 97.8% of users agreed or fully agreed with overall satisfaction with the course, with the highest percentage for the course “Successful Breastfeeding”. 

Spanish language programme version: The highest number of users agreeing or fully agreeing that the course has increased their understanding of the topic was seen for the “Human Milk Oligosaccharides” course (99.4%), with the lowest percentage giving this rating for the course “LCPUFAs” (97.3%) (Table 3). The course “Human Milk Oligosaccharides” received the best scoring among all courses for the criterion “The course matched the prescribed time investment”, with 98.5% of users agreeing or fully agreeing while this percentage was lowest, although still higher than 90%, for the course “Complementary Feeding” (93.4%). The highest percentage of users agreed of fully agreed with overall satisfaction for the course “Human Milk Oligosaccharides” (99.4%).

French language programme version: The highest number of users agreeing or fully agreeing that the course has increased their understanding of the topic was seen for the “Successfully Breastfeeding” and “Human Milk Oligosaccharides” courses (98.7%), with the lowest percentage giving this rating for the course “Assessing Child Growth and Development” (88.5%) (Table 3). The course “Successful Breastfeeding” received the best scoring among all courses for the criterion “The course matched the prescribed time investment”, with 90.6% of users agreeing or fully agreeing while this percentage was lowest, for the course “LCPUFA” (80.0%). The highest percentage of users agreed of fully agreed with overall satisfaction for the course “Successful Breastfeeding” (97.5%).

The evaluation results indicated that, overall, the users were highly satisfied with the knowledge gain from the courses and the time investment, while the rating of the users of the French programme version was slightly lower. 

### 3.3. Explorative Evaluation: Open Text Fields

The results for the critical feedback items and suggestions for improvement in the English language evaluation questionnaires show that the themes “Content suggestions or more examples”, “Increasing interactivity or audiovisuals (AV)”, “Technical (quality) issues or problems” were the top three predominant topics addressed for all three courses analyzed. While the first theme was three-times more predominant in the “Successful Breastfeeding” compared to the “Complementary Feeding” course, the request for additional contents or more examples was lowest for the “Successful Breastfeeding” course. Full details are given in Table 4. 

## 4. Discussion

E-learning has become a widely used method for training to enhance knowledge, skills, motivation and attitudes amongst healthcare workers, with increasing acceptance and satisfaction by users during recent years [16]. The evaluation results reported here show a high level of learner completion and satisfaction. The completion and dropout statistics of the ENS programme reported here show that users tend to discontinue the e-learning programme mostly at the beginning of the programme; however, with a low total dropout rate of 6.1% between the first and second course decreasing to only 2.3% between the penultimate and the last course [17]. The dropout rates were only 1.6% from course four to five, which appears to reflect a high motivation of learner’s motivation to continue the programme once they have completed the initial phase. When comparing users successfully passing a CME test with those enrolling to the subsequent course, even users not passing the CME test continue the programme (e.g., failure-rate in the CME test of the first course = 10.8%, while the dropout rate to subsequent course two is only 6.1%). However, the higher the failure rate of a course, the higher the dropout rate for continuing with the next course is. Dropout rates can be influenced by different factors: from personal factors which are not related to the programme such as time constraints, lack of motivation to continue due to difficulty of the content or the tests, or due to dissatisfaction with the programme or course design [6]. Since results concerning the latter two criteria are very good already in the first course (very high completion and learner satisfaction rates), we assume that dropout rates are markedly influenced also by personal and work factors of the learner, such as limited available time for learning or pressure at work for other priorities. While the format and general content of the ENS courses seemed to meet the interest and learning needs of participants to a great extent, thematic analysis of critical and suggestive user feedback has shown that the level of audio-visual-based learning with a focus on videos, interactivity, specific contents and more examples as well as technical (quality) and platform navigation issues should be optimized. For further optimizing the ENS programme, we thus focus on three aspects: reducing even further the minimum learning unit time down to very short stand-alone learning pieces of five to a maximum of ten minutes each, technically fully optimizing all content to smartphone usability to allow for even more location-independent learning, and to increase the percentage of interactive, video-based learning elements focusing on storytelling and case-based scenarios to allow for a maximum of identification of the learner with the content. In-depth learning analytics studies will be designed and implemented for the next cycles of the ENS programme to identify the most effective didactic elements and to develop case-based learning scenarios with national and regional relevance to the learner’s healthcare environments. 

Findings from our other e-learning programmes and platforms such as the Early Nutrition eAcademy Global and the Early Nutrition eAcademy Southeast Asia [18] have shown that customization of the learning content to the needs of the individual learner, regarding composition or level of detail of content by so-called mass customization approaches to e-learning [19,20,21] is an important success factor of e-learning initiatives. Offering the option to create customized and personalized learning curricula allows for a maximum of intrinsic motivation and relevance of learning contents to the learner’s immediate needs. Since this approach requires a highly elaborate IT infrastructure and yet has to be further tested and developed before using in a CME-accredited setting, the immediate next steps for the ENS programme are above-mentioned optimizations and the additional generation of an advanced ENS programme version with specialized content as a next qualification level to participants of the ENS programme.

## 5. Conclusions

We are unaware of any other non-commercial CME-accredited professional qualification e-learning programme for international HCPs such as the ENS programme hosted by a public university and characterized by an unbiased scientific evidence approach to the specialized topic of early nutrition and lifestyle. Evaluation of the ENS programme over three years shows a high level of user satisfaction regarding the quality and relevance of the contents and the learning design offered, and a need for filling this gap in CME. During the recent SARS-CoV2 pandemic, the relevance of such e-learning programmes, which do not require any travel or meeting activities, became even more important. Although pure e-learning has limitations, and blending e-learning with face-to-face teaching may be desirable, e-learning can provide barrier-free global teaching of the healthcare and nutrition community and hence has enormous potential to help tackle the double burden of disease and to improve health in our next generations worldwide.

## Figures and Tables

**Table 1:** Demographic characteristics of registered participants on the Early Nutrition Specialist Programme (ENS) e-learning platform.

Demographics	Number of Participants (*n*) (Total = 4628)	%
Profession		
Dietician	146	3.2%
General Practitioner	237	5.1%
Midwife	17	0.4%
Nurse	76	1.6%
Nutritionist	77	1.7%
**Paediatrician**	**3872**	**83.7%**
Other		4.4%
Gender		
Male	2308	49.9%
Female	2290	49.5%
Other/n.d.	30	0.7%
Age		
18–25	16	0.4%
26–35	845	18.2%
**36–45**	**1868**	**40.4%**
46–55	1036	22.4%
56–65	460	9.9%
66+	59	1.3%
n.d.	344	7.4%
Level of experience		
0–5 years	541	11.7%
5–10 years	1082	23.4%
10–15 years	1160	25.1%
**>15 years**	**1815**	**39.2%**
n.d.	30	0.7%

The highest values are displayed in bold.

**Table 2:** Completion and Continuing Medical Education (CME) test passing rates/dropout rates per course.

Course	Number of Enrolled Users (*n*)	Number of Users Successfully Passed CME Test (*n*)	Dropout Rate from Previous Course (%)	Passing ^1^ and [Failure] Rate (%)
1. Successful Breastfeeding	4003	3569	/	89.2 (10.8) %
2. Assessing Childhood Growth and Development	3758	3537	6.1%	94.1 (5.9) %
3. Complementary Feeding	3630	3452	3.4%	95.1 (4.9) %
4. LC-PUFAsS (Long Chain Polyunsaturated Fatty Acids)	3557	3399	2.0%	95.6 (4.4) %
5. Breast Milk Substitutes	3500	3283	1.6%	93.8 (6.2) %
6. Human Milk Oligosaccharides	3421	3310	2.3%	96.8 (3.2) %

^1^ Passing rate = number of users successfully completing the CME test/number of users with at least one CME test attempt.

**Table 3:** Percentage and number of users responding with the answer “I (strongly) agree” to three selected evaluation questions for the six courses. The answer options offered followed a Likert element with five rating categories (I strongly agree; I agree; I neither agree nor disagree; I disagree; I strongly disagree).

Course	Item 1: The Course has Increased my Understanding of the Topic	Item 2: The Course Matched the Prescribed Time Investment	Item 3: Overall I am Satisfied with this Course
**1. Successful Breastfeeding**			
English users (*n =* 3915)	**99.1**% (*n =* 3878)	94.1% (*n =* 3684)	**97.8**% (*n =* 3828)
Spanish users (*n =* 411)	98.5% (*n =* 405)	95.6% (*n =* 393)	97.8% (*n =* 402)
French users (*n =* 159)	**98.7**% (*n =* 157)	**90.6**% (*n =* 144)	**97.5**% (*n =* 155)
**2. Assessing Childhood Growth and Development**			
English users (*n =* 3771)	**94.6**% (*n =* 3568)	95.7% (*n =* 3609)	95.7% (*n =* 3609)
Spanish users (*n =* 393)	94.4% (*n =* 371)	95.2% (*n =* 374)	96.4% (*n =* 379)
French users (*n =* 157)	**88.5**% (*n =* 139)	88.5% (*n =* 139)	94.3% (*n =* 148)
**3. Complementary feeding**			
English users (*n =* 3686)	97.2% (*n =* 3581)	**91.1**% (*n =* 3358)	95.5% (*n =* 3521)
Spanish users (*n =* 378)	98.4% (*n =* 372)	**93.4**% (*n =* 353)	95.8% (*n =* 362)
French users (*n =* 156)	94.9% (*n =* 148)	80.8% (*n =* 126)	93.0% (*n =* 145)
**4. LC-PUFAs (Long Chain Polyunsaturated Fatty Acids)**			
English users (*n =* 3618)	97.4% (*n =* 3524)	94.1% (*n =* 3404)	96.6% (*n =* 3495)
Spanish users (*n =* 366)	**97.3**% (*n =* 356)	96.2% (*n =* 352)	97.5% (*n =* 357)
French users (*n =* 155)	90.3% (*n =* 140)	**80.0**% (*n =* 124)	92.9% (*n =* 144)
**5. Breast Milk Substitutes**			
English users (*n =* 3540)	98.2% (*n =* 3475)	95.3% (*n =* 3375)	97.4% (*n =* 3448)
Spanish users (*n =* 354)	98.9% (*n =* 350)	96.9% (*n =* 343)	98.9% (*n =* 350)
French users (*n =* 152)	92.8% (*n =* 141)	79.6% (*n =* 121)	92.1% (*n =* 140)
**6. Human Milk Oligosaccharides**			
English users (*n =* 3470)	98.1% (*n =* 3405)	**96.2**% (*n =* 3337)	97.3% (*n =* 3375)
Spanish users (*n =* 342)	**99.4**% (*n =* 340)	**98.5**% (*n =* 337)	**99.4**% (*n =* 340)
French users (*n =* 152)	**98.7**% (*n =* 150)	88.8% (*n =* 135)	94.7% (*n =* 144)
**Mean (all courses)**			
English users	97.4%	94.4%	96.7%
Spanish users	97.8%	96.0%	97.6%
French users	94.0%	84.7%	94.1%

Highest values are displayed in bold.

**Table 4:** Critical feedback and suggestions for improvement clustered in themes given in the open text fields of the English language evaluation questionnaires in each course and their prevalence in numbers (*n*) and % (defined by number of critical or suggestive user comments referring to a theme / total number of critical or suggestive user comments). Highest values are displayed in bold.

	Number of User Comments (*n*) Referring to a Theme and [%]			
Theme	Course 1. Successful Breastfeeding	Course 2. Assessing Childhood Growth and Development	Course 3. Complementary Feeding	Total
(Clinical) case studies	4	42	5	51
Content suggestions or more examples	**85 (17%)**	**169 (25%)**	**175 (42%)**	**429 (34%)**
Course format	28	36	49	113
Increasing interactivity or more AV	**197 (39%)**	**113 (23%)**	**64 (15%)**	**374 (31%)**
Mobile friendliness	19	2	4	25
Offline written course material	28	35	33	96
Technical (quality) issues or navigation problems	**107 (21%)**	**70 (14%)**	**63 (15%)**	**240 (20%)**
Too long	40	21	20	81
Total number of *critical or suggestive* user comments (*n*)	508	488	413	1409
Total number of user comments	3916	3773	3686	11,375

## Data Availability

Reports on descriptive and explorative data analysis can be found on the ENS programme website and upon request per email to the autors.

## Appendix A

**Table A1:** Evaluation Questions, Format and Scaling.

Evaluation Questionnaire: Learner Satisfaction Measure Scale
The course has increased my understanding of the topic. ^1^I feel the course was sufficiently interactive. ^1^
How enjoyable did you find the course? ^2^
How would you rate the overall presentation and design of this course? ^3^
Would you recommend this course to a colleague? ^4^
How well do you feel you performed in the quizzes and interactive questions in the course? ^5^
Did you find the audio within the image hotspots beneficial? ^6^

Note: ^1^ Five-point Likert element (one being strongly disagree and five being strongly agree). ^2^ Rated from one to ten (one was the minimum score (very unenjoyable) and ten the maximum (very enjoyable). ^3^ Rated from one to ten (one was the minimum score (poor) and ten the maximum (excellent). ^4^ Yes/No. ^5^ Five-point Likert scale (one being very well and five being very poorly). ^6^ Yes/No/I didn’t listen to it.
